# The impact of COVID-19 pandemic on suicides in portugal during the emergency state

**DOI:** 10.1192/j.eurpsy.2021.279

**Published:** 2021-08-13

**Authors:** S. Freitas Ramos, M.I. Fonseca Marinho Vaz Soares, J. Martins Correia, B. Jesus, D. Cruz E Sousa, J. Mendes

**Affiliations:** Department Of Psychiatry And Mental Health, Local Health Unit of Guarda, Guarda, Portugal

**Keywords:** Suicide, COVID-19

## Abstract

**Introduction:**

The mental health impact of the COVID-19 pandemic is well documented. Portugal entered the emergency state on 19^th^ march due to rising numbers of infected patients. The emergency state introduced regulatory measures that restricted people’s movements, applied a curfew, and closed most non-essential spaces and activities, such as shops and religious celebrations.

**Objectives:**

To evaluate the rates of suicides during the emergency state in Portugal.

**Methods:**

We obtained the number of probable suicides during 19^th^ march and 2^nd^ may 2020, 2019 and 2018 from SICO/eVM (Real Time Mortality Electronic Surveillance). This system is used for health planning in Portugal and provides provisory data which is updated every 10 minutes. ExcelÒ was used for the statistical analysis.

**Results:**

During the Emergency State in Portugal there were 57 probable suicides. Comparing to the same period in 2018 and 2019, there were 62 and 70 probable suicides, respectively. Social isolation, anxiety, fear of contagion, chronic stress, and economic difficulties may lead to the development or exacerbation of depressive, anxiety, substance use, and other psychiatric disorders. Literature on suicides due to COVID-19 mention not only fear of infection, but also social isolation and distancing and economic recession as causes for suicide attempts and completions.

**Conclusions:**

During the emergency state there was not an increase of probable suicides, compared to previous years. The greater vigilance of people’s movements may have deterred many attempts. However, policymakers and health care providers must be alert as the current psychosocial predispose to an increase in suicide rates.

**Disclosure:**

No significant relationships.

